# Capsaicin supplementation prevents western diet-induced hyperleptinemia by reducing endoplasmic reticulum stress in apolipoprotein E-deficient mice

**DOI:** 10.29219/fnr.v67.9610

**Published:** 2023-12-06

**Authors:** Hyun Ju Kim

**Affiliations:** Kimchi Functionality Research Group, World Institute of Kimchi, Nam-Gu, Gwangju, South Korea

**Keywords:** endoplasmic reticulum stress, capsaicin, Western diet, hyperleptinemia, apolipoprotein E-deficient mice

## Abstract

**Background:**

Endoplasmic reticulum (ER) stress implicated in leptin resistance in the diet-induced obesity, which can accelerate the development of atherosclerosis forms the background of this study.

**Objective:**

This study aimed to investigate the effect of capsaicin on hyperleptinema by inhibiting ER stress in apolipoprotein E-deficient (ApoE^-/-^) mice fed a western diet (WD).

**Design:**

ApoE ^-/-^ mice were assigned one of three experimental diets: WD (60% kcal from fat, *n* = 10), WD + 0.015% capsaicin (*n* = 10, w/w), and WD + 1% PBA (*n* = 10, w/w) for 12 weeks.

**Results:**

In metabolic parameters, supplementation of dietary capsaicin displayed marked reduction of body weight gain and adipose tissue weight, plasma leptin, total cholesterol, and hepatic triglyceride levels without change in the plasma insulin level compared with WD fed ApoE-/- mice after 12 weeks. Capsaicin supplementation also attenuated the protein expression of ER stress markers such as eukaryotic translational initiation factor 2α and C/EBP homology protein in the liver, as well as glucose-related protein 78 localization in the aorta, indicating that capsaicin inhibits diet-induced hyperleptinemia in part by regulating the protein expression involved in ER stress.

**Conclusion:**

Capsaicin, therefore, may have potential as a therapeutic agent for individuals with diet-induced hyperleptinemia.

## Popular scientific summary

- ER stress is implicated in the development of leptin resistance and activation of the UPR results in blockade of the leptin signaling.
- Capsaicin supplementation showed reduction of body weight gain and adipose tissue weight accompanied with reduction of hyperleptinemia in apoE-/- mice fed a WD.
- The results indicate that capsaicin alleviates diet-induced hyperleptinemia in part by inhibiting ER stress.

Atherosclerosis is featured by the accumulation of lipid-rich atheromatous plaques in the arterial wall (1). An increase in atherosclerotic lesions has prominent aspects including lipid accumulation in endothelial cells, infiltration of macrophages, recruitment and transmigration of smooth muscle cells, and gathering of local inflammatory mediators (2). Excess free cholesterol (FC) deposition in macrophages by hypercholesterolemia results in endoplasmic reticulum (ER) stress, triggering the unfolded protein response (UPR), which is present in all stages of progression of atherosclerotic lesions (3). While ER stress is involved in the instability of rupture and thrombosis of atherosclerotic plaques, the UPR is regarded as a new adaptive response to the life or death of cells (4).

ER dysfunction is implicated in many pathophysiological conditions, such as obesity, diabetes, inflammation, hypercholesterolemia, oxidative stress, and ischemia, among others (4). Accumulation of FC in macrophage and formation of reactive nitrogen species in the human vascular endothelium are considered important processes in atherosclerotic lesion formation (5, 6). The accumulation of unfolded proteins by oxidized lipids in the ER induces UPR in endothelial cells and is associated with the activation of ER stress pathways, such as the phosphorylation of eukaryotic initiation factor 2α (eIF2α) and expression of X-box binding protein (XBP)1 and C/EBP homologous protein (CHOP), which results in apoptosis and homeostatic regulation in the ER (7). The 78-kD glucose-regulated/binding immunoglobulin protein (GRP78/BiP) is an ER molecular chaperone that plays a pivotal role in regulating the transcription of ER stress sensors and transducers. Upon ER stress, GRP78/BiP binds unfolded proteins, thereby activating ER stress and triggering the UPR. This results in stabilization of protein folding, which protects cells from prolonged or severe ER stress (3, 8).

Capsaicin is a natural phytochemical compound found in chili peppers, the consumption of which has increased worldwide (9). Consumption of foods containing capsaicin confers many health benefits, including decreased inflammation, hypercholesterolemia, and adipose tissue size and improvement in metabolic diseases (10, 11). However, it is still unclear whether capsaicin supplementation can mitigate hypercholesterolemia and whether it may slow the progression of atherosclerotic lesions by reducing ER stress. To address these questions, we examined whether dietary capsaicin can attenuate hyperleptinemia-induced ER stress responses. Our experiments exhibited that capsaicin attenuates hyperleptinemia-induced ER stress responses concomitantly with decreased adipose tissue mass and leptin resistance in apolipoprotein E deficient (ApoE^-/-^) mice fed a western diet (WD).

## Materials and methods

### Materials

Capsaicin, 4-phenylbutyric acid (PBA), and β-actin (A5441) antibody were from Sigma-Aldrich (St. Louis, MO, USA). Plasma glucose, total cholesterol (TC), and triglyceride (TG) kits were from Asan Pharm Co., Ltd. (Seoul, South Korea) and measured following the manufacturer’s instructions. Plasma levels of interleukin (IL)-6, tumor necrosis factor (TNF)-α, and leptin were measured by enzyme-linked immunosorbent assay (ELISA) kits from Abcam (Cambridge, UK). The ELISA kit for the measurement of insulin levels was purchased from ALPCO Diagnostics (Salem, NH, USA). Primary antibodies for phoshpo-eIF2a (#3398), eIF2a (#2103), XBP1 (#27901), and CHOP (#2895) were from Cell Signaling Technology (Danvers, MA, USA). GRP78 (sc-13539) and secondary antibodies (sc-2357) were obtained at Santa Cruz Biotechnology (Dallas, TX, USA).

### Animals and diets

All procedures were performed according to guidelines approved by the Institutional Animal Care and Use Committee of the Korea Food Research Institute. This study was approved by the Experimental Ethics Committee of Korea Food Research Institute (Approval number: KFRI-M-12030) and the ARRIVE guidelines. Six week old ApoE^-/-^ (male, 20~25 g) mice were from Jackson Laboratories (Bar Harbor, ME) and maintained under a light–dark (12 h/12 h cycle) and temperature (21–23°C). ApoE ^-/-^ mice were assigned one of three experimental diets: WD (60% kcal from fat, *n* = 10; Research Diet), WD + 0.015% capsaicin (*n* = 10), and WD + 1% PBA (*n* = 10). Supplementation of 0.015% capsaicin in the WD was based on its daily consumption in Asian countries including Korea (12). The experimental diet compositions are shown in Table 1. PBA, a chemical chaperone was used as the positive control in this study (13). Body weight and food intake were recorded weekly and every other day, respectively. At 12 weeks, the mice fasted overnight and were sacrificed by CO_2_ asphyxiation. The blood was collected by cardiac puncture and stored in a heparin tube. After centrifugation 1,200×*g*, 10 min, room temperature, plasma was aliquoted and organs were stored at −80°C. The organs were isolated and fixed in 10% buffered neutral formalin for histological analysis.

**Table 1:** Diet composition of experiment (g/kg diet)

Composition	ApoE-/- + WD		
	WD	0.015% C	1% PBA
Casein	195	195	195
DL-methionine	3	3	3
Corn starch	50	50	50
Maltodextrin 10	100	100	100
Sucrose	341	341	341
Cellulose, BW200	50	50	50
Milk fat, Anhydrous	200	200	200
Corn oil	10	10	10
Mineral mix	35	35	35
Calcium carbonate	4	4	4
Vitamin mix	10	10	10
Choline bitartrate	2	2	2
Cholesterol, USP*	1.5	1.5	1.5
Ethoxyquin	0.04	0.04	0.04
Capsaicin	-	0.15	-
4-phenyl butyric acid	-	-	10
Total	1001.54	1001.69	1011.54
Kcal/kg	4,686	4,686	4,686

### Measurement of biochemical parameters

Plasma glucose, TC, and TG concentrations were determined using commercial kits according to the manufacturer’s instructions. ELISA kits were used to measure the plasma levels of IL-6, TNF-α, leptin, and insulin.

### Hepatic lipid profiling

Hepatic lipids were extracted using the modified method described by Folch et al. (14). Frozen liver tissue (50 mg) was homogenized nine times with 0.9% salt solution, and then 3 mL chloroform-methanol (2:1) were added. The homogenates were left to stand for 2 h before being centrifuged for 15 min at 3,000 × *g*. The organic phase was vaporized under a nitrogen flow and then dissolved in chloroform. The mixtures were solubilized with Triton X-100, and the extracted lipid contents were quantified with enzymatic TC and TG kits.

### Western blot analysis

Western blotting was performed using our previous procedures (15). Total protein concentration was determined by BCA (#23225, Pierce, Rockford, IL). The protein (40 µg) was separated by a 12% SDS-PAGE gel and transferred onto a 0.2 µm immobile PVDF membrane (Bio-Rad Laboratories, Hercules, CA) with transfer buffer (25 mM Tris-HCl (pH 8.9), 192 mM glycine, and 20% methanol). The membranes were cut according to molecular weight range of antibodies and incubated with primary antibodies against p-eIF2a(#3398), eIF2a(#2103), XBP1(#27901), CHOP(#2895) from Cell Signalling (Danvers, MA, USA) at 1:1000, and β-actin(A5441) from Sigma-Aldrich (St. Louis, MO, USA) at 1:10000 dilution overnight at 4°C. The membranes were washed three times, incubated with secondary anti-mouse or anti-rabbit IgG antibodies, and visualized using enhanced chemiluminescence (SYNGENE, Frederick, MD). The relative protein levels of p-eIF2α, eIF2, XBP1, and CHOP were calculated based on the ratio of intensity of each protein bands to the corresponding β-actin. Band densities were quantified using a ImageJ Launcher.

### Histology and immunohistochemistry

Aortic root tissue was fixed with 10% formalin and embedded in paraffin blocks. Blocks were cut into 4-µm thick sections using a rotary microtome, stained with hematoxylin and eosin (H&E) according to standard procedures, and histologically analyzed with a microscope (SV40; Olympus, Tokyo, Japan) at 20 × magnification. Paraffin sections were deparaffinized for immunohistochemical analysis. Aortic sections were treated with 1% H_2_O_2_ and blocked with 5% skim milk in PBS. The sections were incubated with mouse primary anti-GRP78 antibody and stained with an ABC Kit (Vector Laboratories, Burlingame, CA). Immunostaining was detected by a 3, 3’-DAB Kit (Vector Laboratories). Sections incubated with 10% non-immune mouse serum were used as the negative controls.

### Statistical analysis

All data are mean ± standard error of the mean. Statistical analysis was conducted using SPSS version 23 (IBM, Armonk, NY). Data were analyzed using Duncan’s test and statistical significance was established at *P <* 0.05.

## Results

### Capsaicin ameliorates body weight gain and leptin resistance

ApoE^-/-^ mice supplemented with 0.015% capsaicin showed decreased gains in body, liver, abdominal fat, epididymal fat, and brown fat weight. These parameters were reduced by 45%, 10%, 41%, 35%, and 27%, respectively, compared with those of the WD group. Plasma levels of leptin were dramatically reduced by 73% in the capsaicin-supplemented group compared with the WD group (Table 2). Compared with the WD group, levels of plasma TC in the capsaicin group were significantly lower by 18 % (Table 2). Hepatic TG levels in the capsaicin-supplemented group significantly decreased compared with that of the WD group Mice in the capsaicin group showed decreased plasma IL-6 levels compared with the WD group, whereas TNFα levels did not differ significantly. The plasma levels of glucose and insulin were not significantly different between the WD and capsaicin-supplemented groups (Table 2). Therefore, body weight gain and white and brown fat weight were reduced concomitantly with decreased plasma leptin levels in both capsaicin- and PBA-supplemented ApoE^-/-^ mice.

**Table 2:** Effects of capsaicin on body weight gain and fat weight, plasma and hepatic lipids, glucose, insulin, leptin, and cytokines in WD-fed ApoE^-/-^ mice

Group^1^	ApoE-/- + WD		
	WD	0.015% C	1. PBA
Body weight gain (g/day)	0.18 ± 0.01^a^	0.10 ± 0.01^b^	0.15 ± 0.01^a^
Food intake (g/day)	3.06 ± 0.09^aS^	2.54 ± 0.04^a^	3.10 ± 0.09^a^
Food efficacy ratio (%)^2^	5.75 ± 0.42^a^	3.75 ± 0.31^c^	4.89 ± 0.29^b^
Liver weight (g)	4.38 ± 0.31^a^	3.96 ± 0.12^ab^	4.66 ± 0.21^a^
Visceral abdominal fat weight (g)	1.61 ± 0.09^a^	0.96 ± 0.08^b^	1.15 ± 0.09^a^
Epididymal fat weight (g)	3.83 ± 0.22^a^	2.49 ± 0.12^b^	3.05 ± 0.14^ab^
Brown fat weight (g)	0.47 ± 0.05^a^	0.34 ± 0.02^b^	0.33 ± 0.02^b^
Plasma total cholesterol (mg/dL)	714.25 ± 24.09^a^	589.04 ± 35.98^b^	676.70 ± 42.00^ab^
Plasma triglyceride (mg/dL)	92.86 ± 4.29^a^	87.76 ± 10.15^a^	89.04 ± 7.47^a^
Hepatic total cholesterol (mg/g)	10.36 ± 0.57^a^	10.00 ± 0.41^a^	6.66 ± 0.31^b^
Hepatic triglyceride (mg/g)	70.89 ± 10.01^a^	62.90 ± 9.09^ab^	57.70 ± 8.01^b^
Fasting glucose (mg/dL)	154.58 ± 5.09^b^	170.02 ± 11.56^b^	209.32 ± 10.96^a^
Insulin (ng/mL)	0.153 ± 0.002^a^	0.169 ± 0.013^a^	0.162 ± 0.002^a^
Leptin (pg/mL)	24518.7 ± 2712.4^a^	6727.0 ± 697.3^c^	14654.5 ± 2403.7^b^
Interleukin-6 (pg/mL)	5.27 ± 0.87^a^	4.50 ± 0.61^ab^	4.50 ± 0.57^ab^
Tumor necrosis factor - (pg/mL)	34.18 ± 3.21^b^	44.07 ± 6.61^a^	31.64 ± 3.40^b^

Data means the mean ± SEM (*n* = 10 per group). Values with different letters within a column are significantly different from each other at *P* < 0.05 by Duncan’s multiple range test (a > b > c).

1 The apoE^-/-^ mice was fed a western diet (WD) for 12 weeks. The ApoE^-/-^ + WD + 0.015% C or 1% PBA were fed a WD with supplementation of capsaicin 0.015% or PBA 1% for 12 weeks.

2 The food efficacy ratio is expressed as the total weight gain/total food intake.

### Capsaicin modulates the expression of protein levels involved in ER stress in the liver

**Fig. 1:**
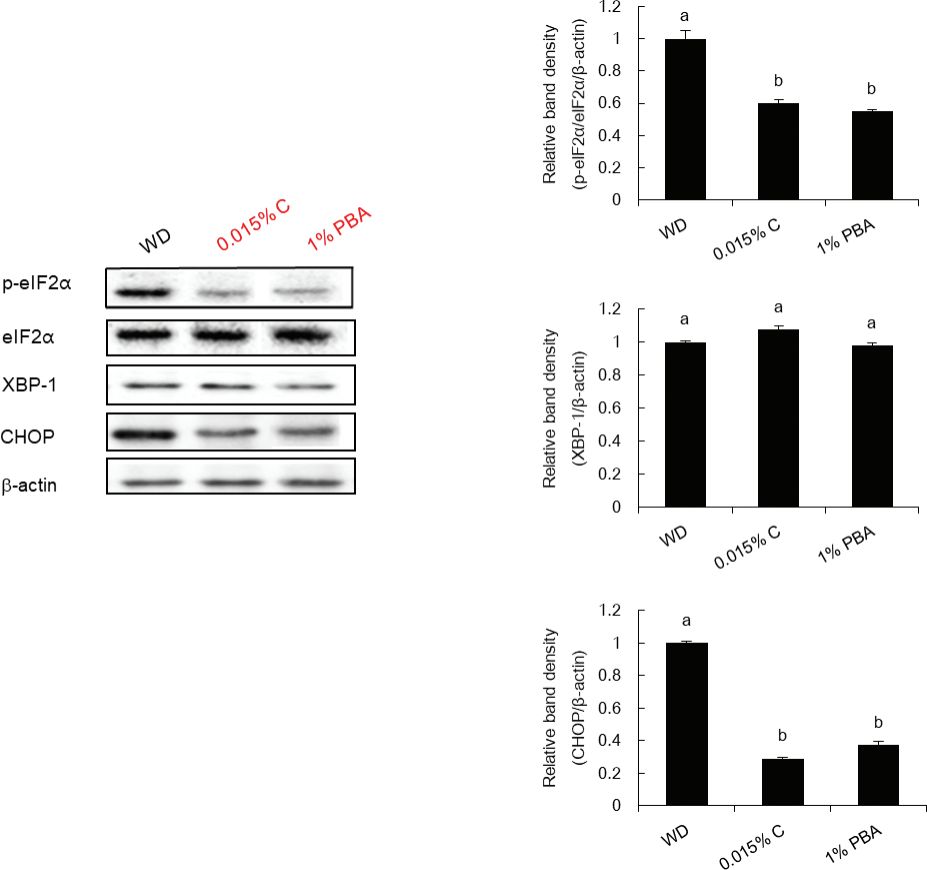
Capsaicin modulates the expression of protein levels involved in ER stress in the liver of ApoE^-/-^ mice fed a WD. Expression of protein levels of p-eIF2α, eIF2α, XBP1, and CHOP were measured by Western blot. Representative western blots and average band intensities of ER stress markers, which was normalized by β-actin. Results are mean ± standard error of the mean (*n* = 4~5 per group). The different letters on the bar represent significant differences from each other at *P* < 0.05. p-eIF2α, phospho-eukaryotic initiation factor 2 subunit alpha; XBP1, X-box binding protein 1; CHOP, C/EBP homologous protein.

To elucidate whether capsaicin inhibited hepatic ER stress, we investigated the protein patterns involved in ER stress in diet-induced hypercholesterolemia models. The protein abundances involved in ER stress, including p-eIF2a and CHOP were increased in the WD-fed ApoE^-/-^ mice group, indicating a state of activated UPR. In contrast, as shown in Fig. 1, the protein abundances of p-eIF2a and CHOP in the livers of capsaicin supplemented mice were significantly lower than those of mice in the WD group. This was accompanied by a drop in hepatic TG content and liver weight, indicating that hypercholesterolemia induced hepatic ER stress in the WD-fed ApoE ^-/-^ mice and that these changes could be reversed by capsaicin supplementation.

### Capsaicin reduces aortic GRP78 expression

**Fig. 2:**
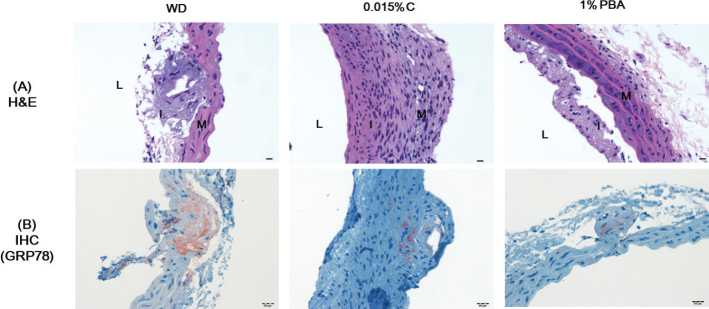
Capsaicin reduces aortic GRP78 expression. Representative Hematoxylin & eosin (a) staining (scale bar, 50 μm, upper panel) and GRP78 immunostaining (scale bar, 20 μm, lower panel) (b) of aortic root sections from ApoE^-/-^ mice fed a western diet. I indicates intima; M, media; and L, lumen. Original magnification, 200 x. GRP78, glucose-regulated/binding immunoglobulin protein-78.

Plaques and marked cell proliferation were detected in the aortic roots of mice in the WD group but not in those of mice in the 0.015% capsaicin- and 1% PBA-supplemented groups (Fig. 2a). GRP78 overexpression in the aortic root was found in mice in the WD group (Fig. 2b). In contrast, there was no observed GRP78 localization in the aortas of capsaicin-supplemented mice when visualized by immunostaining, indicating that hypercholesterolemia-induced ER stress was inhibited by capsaicin supplementation.

## Discussion

Leptin is an anti-obesity hormone which comes from fat that represses appetite and increases energy consumption via its action on the hypothalamus (16, 17). ER stress is also entangled in the development of leptin resistance, and activation of the UPR results in blockade of the leptin signaling network (18). Overnutrition induces hyperleptinemia and hyperinsulinemia by activating the tyrosine kinase Janus kinase (JAK) and the transcription factor signal transducer and activators of transcription (STAT) and IkB kinase (IKK)-β-NFκB signaling pathway through disruption of ER function (18). In addition, ER stress inhibitors improve leptin sensitivity and attenuate obesity by suppressing hypothalamic IKKβ/NF-κB and increasing STAT-JAK as a leptin sensitizers in mice (18–20).

Capsaicin affects energy homeostasis, and glucose metabolism may be involved in the activation of transient receptor potential vanilloid subtype 1 (TRPV1), which is a regulator of leptin signaling (21–24). PBA, an inhibitor of ER stress, increases the activity of brown adipose tissue (BAT) and glycolysis-related genes. This is consequently accompanied by a decrease in BAT weight in high fat diet-induced obesity (25). In addition, capsaicin and capsinoids prevent obesity by increasing energy expenditure and activating the TRPV1–sympathetic nervous system–BAT axis (24, 26). TRPV1 can be activated by piquant compounds, including fermented foods (*Lactobacillus*), cruciferous vegetables (sulforaphane), garlic (allicin), red peppers (capsaicin), and ginger (gingerol). Activation of this receptor may have a significant effect on obesity and can inhibit ER stress (27). In our study, supplementation with capsaicin ameliorated leptin resistance and adipose tissue weight gain, whereas no modification in plasma insulin and glucose levels were observed (Table 2), suggesting that capsaicin intake may lead to improvements in WD-fed ApoE ^-/-^ mice with hypercholesterolemia and hyperleptinemia by increasing BAT energy expenditure. Capsaicin markedly upregulated genes involved in glucose metabolism and modulated gut microbiota by activating the TRPV1, indicating the anti-obesity of capsaicin (28–30). Furthermore, the hypoglycemic effect of capsaicin was mediated by upregulating TRPV1-pancreatic duodenal homebox-1 signaling pathways in the liver of diabetic rats (31). Unexpectedly, capsaicin supplementation caused slight increase of fasting glucose level in plasma, although not significant between the WD and capsaicin treated groups. Despite accumulated evidence showing that capsaicin has a positive effect on glycometabolism in the cell and animal models, systemic review from human studies pointed out the lack of effects on glucose and insulin levels (32).

The ER is a crucial organelle of lipid synthesis, assembly, and droplet formation; ER function is compromised in the accumulation of excess lipids, leading to ER stress (33–35). In our study, hepatic TG levels were slightly decreased by capsaicin. This may be associated with XBP1 protein expression in the liver, suggesting that the inositol-requiring enzyme type 1 (IRE1) branch of the UPR pathway was induced (Table 2 & Fig. 1). XBP1, which is found downstream of IRE1α, regulates hepatic lipogenic and glycolytic genes (36). Despite this decrease, markers involved in lipogenesis were not detected in the liver in our study.

ER stress-CHOP-mediated apoptosis is triggered by the accumulation of FC in infiltrating macrophages within atherosclerotic lesions (37). Oxidized lipids are implicated in ER stress induction and triggers phosphorylation of IRE1α and eIF2α, which can be prevented by antioxidants. These results are in parallel with other studies revealing that 7-ketocholesterol induces apoptosis of vascular cells via the activation of the IRE1 pathway (8). Liver XBP-1 and eIF2α depletion is accompanied by decreased hepatic steatosis in obese animals (36, 38). Together, our results indicate that the decrease in liver ER stress markers by capsaicin supplementation leads to a clear improvement of hypercholesterolemia in WD-fed ApoE^-/-^ mice.

In our previous study, 7-ketocholesterol induced a marked increase of ER stress markers and cell death in macrophages (6). Several studies have showed that oxidized low-density lipoprotein and lipid peroxidation products trigger ER stress and the production of UPR markers, featured by observations of GRP78 localization in ApoE^-/-^ mouse vascular cells and atherosclerotic lesions (3, 8) and in the plasma of people with metabolic disorders and atherosclerosis (39). In addition, Zhou et al. (3) observed not only the overexpression of ER stress inducers, FC, and peroxynitrite, but also ER stress markers like GRP78, calreticulin, CHOP, and pPERK in early-stage atherosclerotic lesions. GRP78 expression has been shown to alleviate fatty liver diseases by suppression of ER stress-induced lipogenesis in obese mice (40).

Our results demonstrate that dietary supplementation with capsaicin can significantly alleviate hypercholesterolemia as well as hyperleptinemia, which is accompanied with a marked decrease in adipose tissue weight in WD-fed ApoE^-/-^ mice. Moreover, we showed that capsaicin alleviates the rates of atherosclerotic lesions in diet-induced hypercholesterolemia by inhibiting the expression of ER stress proteins like eIF2α, XBP-1, CHOP, and GRP78. These results reveal that capsaicin may be a potent dietary agent for preventing diet-induced hypercholesterolemia and cardiovascular disease. However, further research is needed to explore the fundamental effects of capsaicin on the mechanism of energy metabolism in the central metabolic organ in obesity-related metabolic diseases.

## Data Availability

Data are all contained within the article.
